# Reply To: Comments on identifying causal relationships in nonlinear dynamical systems via empirical mode decomposition

**DOI:** 10.1038/s41467-022-30360-1

**Published:** 2022-05-23

**Authors:** Albert C. Yang, Chung-Kang Peng, Norden E. Huang

**Affiliations:** 1 Institute of Brain Science/Digital Medicine and Smart Healthcare Research Center, National Yang Ming Chiao Tung University, Taipei, Taiwan; 2 Department of Medical Research, Taipei Veterans General Hospital, Taipei, Taiwan; 3 Division of Interdisciplinary Medicine and Biotechnology, Beth Israel Deaconess Medical Center/Harvard Medical School, Boston, MA 02215 USA; 4 Key Laboratory of Data Analysis and Applications, First Institute of Oceanography, SOA, Qingdao, 266061 China

**Keywords:** Ecological modelling, Applied mathematics

**replying to** Chun-Wei Chang et al. *Nature Communications* 10.1038/s41467-022-30359-8 (2022)

The preceding Matters Arising^1^ on our Article^2^ states that (1) empirical mode decomposition (EMD) method incorrectly distinguish causation from correlation for the system of two independent variables driven by a shared external forcing (aka Moran effect^3^); and (2) we used convergent cross mapping (CCM) in a manner that was not intended by the original paper^4^ and that leads to incorrect causal relationship interpreted by the CCM method. While we think the second comment has certain merits, the first point needs to be addressed in detail.

First, Chang et al.^1^ criticized that causal decomposition fails to correctly identify causal relationships in adult-recruitment model to simulate Moran effect in which two independent variables are driven by a shared external force. Here we reach a different conclusion, which we now elaborate. It’s obvious that *N*_*1*_ and *N*_*2*_ in the adult-recruitment model are correlated because of the common environmental variable in the differential equation. However, with appropriate mathematical deduction (see supplementary information), both the values of *N*_*1*_ and *N*_*2*_ are found to be coupled with the past values of its counterpart, which accords temporal precedence principle of cause and effect. Furthermore, we found the causal strength between *N*_*1*_ and *N*_*2*_ was mainly driven by the ratio of *φ*_1_ and *φ*_2_, which represents the magnitude of environmental forcing in the adult-recruitment model. Fig. 1a shows that a change in relative causal strength by causal decomposition is observed in various settings of *φ*_1_ and *φ*_2_. Interestingly, a consistent finding of convergence of cross mapping between *N*_*1*_ and *N*_*2*_ is also observed with the CCM method (Fig. 1b), suggesting a bidirectional coupling exists between *N*_*1*_ and *N*_*2*_, which contradicts the conclusion by Chang et al.^1^ that CCM would indicate no causation in this model.

**Fig. 1:**
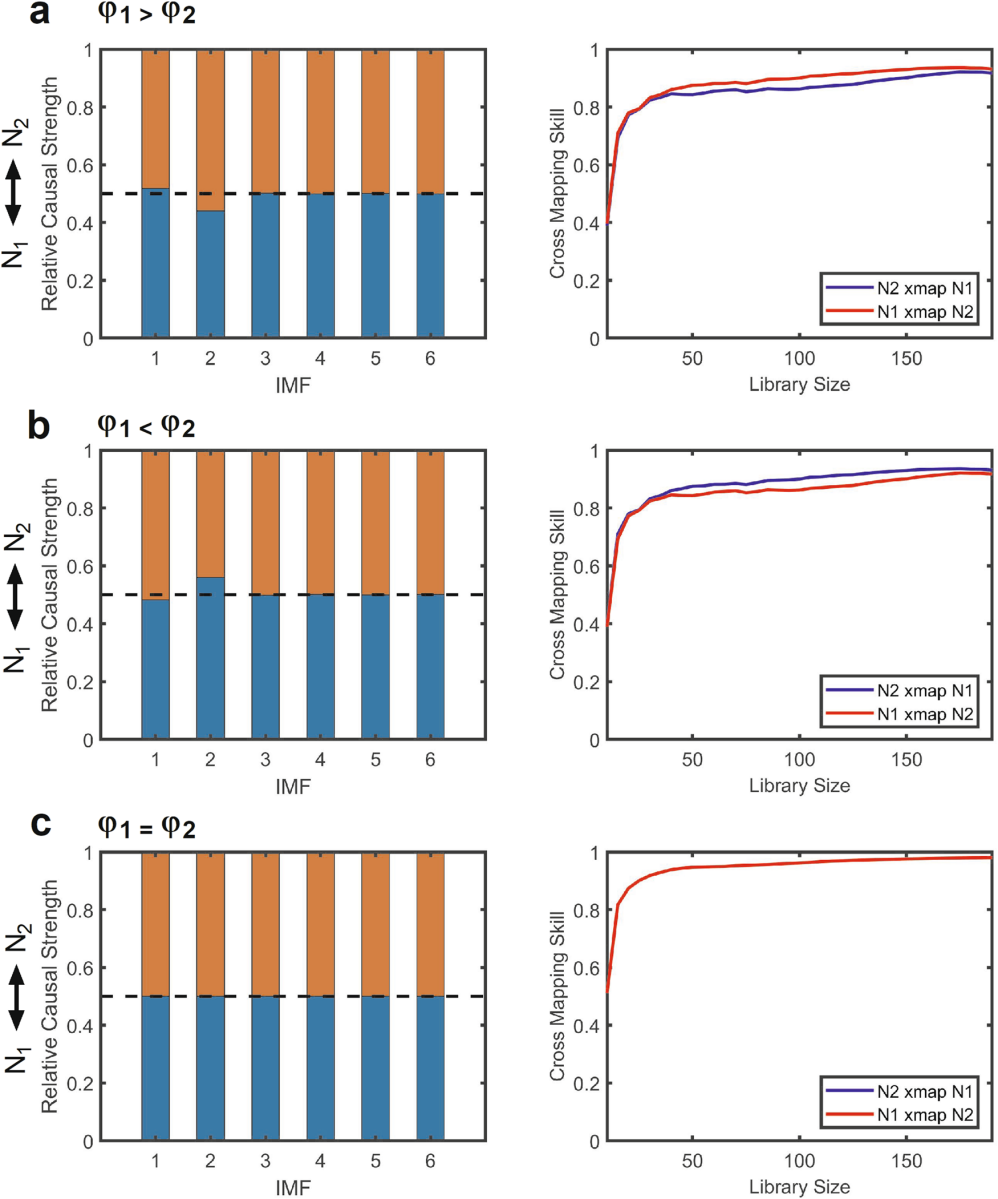
Causal patterns of adult-recruitment model in various settings of environmental forcing. The adult-recruitment model is a 5-variate differential equation to simulate the Moran effect that the populations of two independent species N_1_ and N_2_ are driven by the external forcing of the environmental noise. With appropriate mathematical deductions (see supplementary information), we found that N_1_ and N_2_ are causally coupled with each other and the causal strengths between them are driven by the ratio of environmental forcing. For simplicity, the parameters were given here as *r*_*1*_ = 3.4, *s*_*1*_ = 0.4, and *D*_*1*_ = 3, as well as *r*_*2*_ = 3.4, *s*_*2*_ = 0.4, and *D*_*2*_ = 3. The initial value of *R* and *N* was set as 1 and 0.5, respectively in both pairs. The model was simulated for 10,000 iterations and the last 200 data points of N_1_ and N_2_ were extracted for causal analysis with causal decomposition and convergent cross mapping (CCM) method. The parameters for causal decomposition and CCM method were the same with Chang et al^1^. (i.e., noise level = 0.085, ensemble number = 1000, and embedding dimension for CCM = 4). We tested the causal decomposition and CCM results in the various settings of environmental forcing at (**a**), *φ*_1_ = 0.6 and *φ*_2_ = 0.5; (**b**), *φ*_1_ = 0.5 and *φ*_2_ = 0.6; and (**c**), *φ*_1_ = 0.5 and *φ*_2_ = 0.5. As expected, the causal decomposition showed a correct direction of coupling in these different settings of environmental forcing. CCM also showed a consistent pattern of bi-directional coupling between N_1_ and N_2_ in three settings (statistical tests of Kendall’s τ test and Fisher’s Δρ Z score for the significance of convergence of cross mapping showed *p* < 0.05 in all settings.).

It is worthy to note that the Moran effect model may be a case of instantaneous causality^5^. The example illustrated by Chang et al.^1^ indicates that CCM accounts for instantaneous causality as the states of the variables compared are at the same time point and therefore shows no causality, whereas the result of the causal decomposition on the other hand indicated lag-causality instead of instantaneous causality. The presence of instantaneous causality in the nonlinear system such as Moran effect models requires further study^6^. Additionally, both CCM and causal decomposition analysis are bivariate causality analyses, common drivers and other indirect causal effects may be better identified using multivariate causality approaches^7,8^.

Second, we appreciate Chang’s et al.^1^ clarification of the importance of the convergence of cross mapping in the interpretation of the method. This criterion gives clue to the existence of causation but do not indicate the difference in coupling strengths such as top-down or bottom-up control in the predator and prey relationship. For clarity, here we quote the original statement for the CCM results of *Didinium* and *Paramecium* by Sugihara et al.^4^: “*The results in Didinium and Paramecium suggest bidirectional coupling, which accords with what is known. Moreover, the higher level of skill in cross mapping Didinium from the Paramecium time series than the reverse suggests that top-down control by the predator, Didinium, is stronger than bottom-up control by the prey, Paramecium*.” Therefore, it seems to us that the interpretations of CCM are two folds: (1) the existence of directional coupling is determined based on the convergence or improvement of cross-mapping skills^4,9^, and (2) the strength of coupling in each direction (e.g., top-down or bottom-up control in the case of predator and prey) is determined by difference in the level of cross-mapping skill (e.g., correlation)^4^.

Based on these definitions, we did have objective interpretation of CCM results in Fig. 5 of our Article^2^ that all ecology data showed bi-directional coupling but only *Didinium* and lynx have a clear pattern of top-down control, whereas CCM failed to show differential control in Lotka Volterra model and the convergence of cross-mapping skill is ambiguous in wolf-moose relationship. In contrast, the causal decomposition consistently identifies top-down control of predator over prey across four types of ecology data. As already mentioned in our paper, the relative causal strength is more important than absolute causal strength in that a meaningful causality is only observed when differential coupling strengths exist. Having that said, the interpretation of CCM by McCracken et al.^10^ using difference of cross-mapping skill is consistent with Sugihara et al.^4^. But we do concur that the convergence criteria of cross-mapping skill needs to be rigorously evaluated for the presence of causation^9^.

For additional comments by Chang et al.^1^, we concur that CCM does not rely on predictability but information recovering as the criterion. However, we would like to highlight that the ability of information recovering in state space is determined by the choice of embedding dimension, which is related to the length of time lag in time series model. Furthermore, there indeed have been methods to improve CCM with trend removing techniques, and we have discussed its pros and cons in our published Peer Review File^11^.

Chang et al.^1^ also commented that causal decomposition does not meet the expectation that if the predator dies off exponentially in the absence of the prey, then the prey will grow exponentially in the absence of the predator; rather, the remaining components continue to cycle after subtracting causal IMFs. We would like to point out that the causal decomposition method was not intended to mathematically solve the differential equation of predator and prey model. Specifically, although we would expect the predator and prey will have respectively exponential decay or growth in the absence of the other pair, it’s apparent that removing a causal-related IMF in a time series will not induce exponential change as predicted by the predator and prey model because the data has been recorded in a manner where both predator and prey are present. Furthermore, because EMD is designed to separate oscillations in different temporal scales^12^, the method itself does not necessarily violate the mathematical intuition of non-separability given in the nonlinear differential equations. For example, we have shown that EMD method is able to delineate phase and amplitude coupling from nonlinear oscillations generated by the multiplicative process^13^.

Importantly, we concur that real-world data is blended with stochastic and deterministic mechanisms. Our paper never intended to disparage Granger or CCM method. We have objectively shown the merit of use of covariation principle of causality, and illustrate advantages and limitations of causal decomposition compared to other methods. Nevertheless, more data and validation are absolutely needed to evaluate the applicability of existing causality methods in various modeled and real-world data.

## Reporting Summary

Further information on research design is available in the Nature Research Reporting Summary linked to this article.

## Supplementary information

Supplementary Information

reporting summary
