# Supplementary material for: Reply To: Comments on identifying causal relationships in nonlinear dynamical systems via empirical mode decomposition
Source: Nat Commun. 2022 May 23;13:2859. doi: 10.1038/s41467-022-30360-1 (PMC9127069; doi:10.1038/s41467-022-30360-1)
Supplement: Supplementary file 1 — Supplementary Information [file 41467_2022_30360_MOESM1_ESM.docx]

**Supplementary Information**

**Reply To: Comments on identifying causal relationships in nonlinear dynamical systems via empirical mode decomposition**

Albert C. Yang^1,2*^, Chung-Kang Peng^3^ and Norden E. Huang^4^

^1^ Institute of Brain Science / Digital Medicine Center, National Yang Ming Chiao Tung University, Taipei, Taiwan

^2^ Department of Medical Research, Taipei Veterans General Hospital, Taipei, Taiwan

^3^ Division of Interdisciplinary Medicine and Biotechnology, Beth Israel Deaconess Medical Center/Harvard Medical School, Boston, Massachusetts, 02215, USA

^4^ Key Laboratory of Data Analysis and Applications, First Institute of Oceanography, SOA, Qingdao, 266061, China

***Corresponding Author:** Dr. Albert C. Yang, M.D., Ph.D.

Present address: Institute of Brain Science / Digital Medicine Center, National Yang Ming Chiao Tung University, Taipei, Taiwan

No. 155 Li-Nung St. Sec. 2 Beitou Dist.

Taipei City, 112, Taiwan.

Email: [accyang@nycu.edu.tw](mailto:accyang@nycu.edu.tw)

The adult-recruitment model is a 5-variate differential equation model to simulate the Moran effect that an environmental white noise variable, *V*, is passed into the two pairs of interacting variables, recruitment (*R_1_*) and adult (*N_1_*), as well as *R_2_* and *N_2_*, respectively. The nonlinear interaction between *R* and *N* is given in Eq. 1.

$R\left( t+1 \right)=r\times N\left( t \right)\times\left[ 1-N\left( t \right) \right]\times e^{-\varphi\times V\left( t \right)}$

$N\left( t+1 \right)=s\times N\left( t \right)+max\left[ R(t-D),0 \right]$ (1)

Here *r*, *s*, $\varphi$, and *D* represent specific parameters for a given pair of *R* and *N.* According to Chang et al., the parameters were given as *r_1_* = 3.4, *s_1_* = 0.4, $\varphi_{1}=0.5$, and *D_1_* = 3, as well as *r_2_* = 2.9, *s_2_* = 0.35, $\varphi_{2}=0.6$, and *D_2_* = 3. The initial value of *R* and *N* was set as 1 and 0.5 respectively in both pairs. The Eq. 1 can be understood that $\varphi$ determines the magnitude of effect of environmental noise *V* on the interaction between *R* and *N*. For a comparable $\varphi_{1}$ and $\varphi_{2}$, the environmental noise *V* will result in synchronization of the oscillation observed in *N_1_* and *N_2_*, but intuitively they do not have direct coupling between them. This adult-recruitment model is used to explain the Moran effect that the correlation between the population of two non-interacting species is driven by a shared component such as environmental forcing.

The concept of Moran effect is well understood in terms of population ecology. However, the intuition of Moran effect may not be consistent with the adult-recruitment model suggested by Chang et al. Here we show that by rewriting Eq. 1, seemingly unrelated variables *N_1_* and *N_2_* can actually interact with each other because of the existence of common variable *V* in the differential equation.

First, we can take logarithm of the first part of Eq. 1, which results in the following Eq. 2 and 3.

$\log R\left( t+1 \right)=\log r+\log N\left( t \right)+\log\left[ 1-N\left( t \right) \right]-\varphi\times V\left( t \right)$ (2)

Then Eq. 2 can be further rearranged as follows:

$V\left( t \right)=\frac{1}{\varphi}\left\{ \log r+\log N\left( t \right)+\log\left[ 1-N\left( t \right) \right]-\log R\left( t+1 \right) \right\}$ (3)

Therefore, for a given set of *R_1_* and *N_1_*, as well as *R_2_* and *N_2._* We can join the different sets of *R* and *N* by Eq. 3 because $V\left( t \right)$ is a common variable.

$$\frac{1}{\varphi_{1}}\left\{ \log r_{1}+\log N_{1}\left( t \right)+\log\left[ 1-N_{1}\left( t \right) \right]-\log R_{1}\left( t+1 \right) \right\}$$

$=\frac{1}{\varphi_{2}}\left\{ \log r_{2}+\log N_{2}\left( t \right)+\log\left[ 1-N_{2}\left( t \right) \right]-\log R_{2}\left( t+1 \right) \right\}$ (4)

From Eq. 4, we can further recover $R_{1}\left( t+1 \right)$ as follows:

$R_{1}\left( t+1 \right)=e^{\left\{ \log r_{1}+\log N_{1}\left( t \right)+\log\left[ 1-N_{1}\left( t \right) \right] \right\}-\frac{\varphi_{1}}{\varphi_{2}}\left\{ \log r_{2}+\log N_{2}\left( t \right)+\log\left[ 1-N_{2}\left( t \right) \right]-\log R_{2}\left( t+1 \right) \right\}}$ (5)

Then Eq. 5 can be further generalized to $R\left( t-D \right)$ shown in Eq. 1.

$R_{1}\left( t-D \right)=e^{\left\{ \log r_{1}+\log N_{1}\left( t-D-1 \right)+\log\left[ 1-N_{1}\left( t-D-1 \right) \right] \right\}-\frac{\varphi_{1}}{\varphi_{2}}\left\{ \log r_{2}+\log N_{2}\left( t-D-1 \right)+\log\left[ 1-N_{2}\left( t-D-1 \right) \right]-\log R_{2}\left( t-D \right) \right\}}$ (6)

Therefore, for a given $N_{1}\left( t+1 \right)$ and $N_{2}\left( t+1 \right)$, they can be rewritten as follows:

$$N_{1}\left( t+1 \right)= s_{1}\times N_{1}\left( t \right)+$$

$$max\left[ e^{\left\{ \log r_{1}+\log N_{1}\left( t-D-1 \right)+\log\left[ 1-N_{1}\left( t-D-1 \right) \right] \right\}-\frac{\varphi_{1}}{\varphi_{2}}\left\{ \log r_{2}+\log N_{2}\left( t-D-1 \right)+\log\left[ 1-N_{2}\left( t-D-1 \right) \right]-\log R_{2}\left( t-D \right) \right\}},0 \right]$$

$$N_{2}\left( t+1 \right)= s_{2}\times N_{2}\left( t \right)+$$

$$max\left[ e^{\left\{ \log r_{2}+\log N_{2}\left( t-D-1 \right)+\log\left[ 1-N_{2}\left( t-D-1 \right) \right] \right\}-\frac{\varphi_{2}}{\varphi_{1}}\left\{ \log r_{1}+\log N_{1}\left( t-D-1 \right)+\log\left[ 1-N_{1}\left( t-D-1 \right) \right]-\log R_{1}\left( t-D \right) \right\}},0 \right]$$

(7)

Although the adult-recruitment model is designed to explain Moran effect that *N_1_* and *N_2_* are correlated but non-interacting, from Eq. 7 it clearly shows that the current values of *N_1_* or *N_2_* can be determined by its own past values*_,_* as well as the past values of their counterpart*_,_* in which the coupling of counterpart on the original variable is driven mainly by the ratio of $\varphi_{1}$ to $\varphi_{2}$. It is noted that parameters *s_1_, r_1_*, *s_2_, r_2_* also determines the changes of *N* value in the iteration process, hence differential coupling strengths between *N_1_ and N_2_* would exist with parameters being set differently in two species. For simplicity, under the condition that parameters r and s are set to be the same in each species of the model, we can expect that when $\varphi_{1}$is greater than $\varphi_{2}$, there is a stronger coupling from *N_2_* to *N_1_*, and vice versa, as demonstrated by Table 1 in the main text using causal decomposition analysis.
